# Exploring CNS Involvement in Pain Insensitivity in Hereditary Sensory and Autonomic Neuropathy Type 4: Insights from Tc−99m ECD SPECT Imaging

**DOI:** 10.3390/tomography9060175

**Published:** 2023-12-18

**Authors:** Cheng-Chun Chiang, Yu-Che Wu, Chiao-Hsin Lan, Kuan-Chieh Wang, Hsuan-Ching Tang, Shin-Tsu Chang

**Affiliations:** 1 Department of Medical Education and Research, Kaohsiung Veterans General Hospital, Kaohsiung 813, Taiwan; jimchiangcc@gmail.com (C.-C.C.); wij840722@gmail.com (Y.-C.W.); 2 School of Medicine, National Defense Medical Center, Taipei 114, Taiwan; chlanpaper@gmail.com (C.-H.L.); bobbywang7261@gmail.com (K.-C.W.); ymes9860117@gmail.com (H.-C.T.); 3 Department of Physical Medicine and Rehabilitation, Kaohsiung Veterans General Hospital, Kaohsiung 813, Taiwan; 4 Department of Physical Medicine and Rehabilitation, Taichung Veterans General Hospital, Taichung 407, Taiwan; 5 Department of Physical Medicine and Rehabilitation, Tri-Service General Hospital, Taipei 114, Taiwan

**Keywords:** hereditary sensory and autonomic neuropathy type 4, single-photon emission computed tomography, postcentral gyrus, thalamus, cerebellum, pain, central nervous system, cerebral blood perfusion

## Abstract

Hereditary sensory and autonomic neuropathy type 4 (HSAN4), also known as congenital insensitivity to pain with anhidrosis (CIPA), is a rare genetic disorder caused by NTRK1 gene mutations, affecting nerve growth factor signaling. This study investigates the central nervous system’s (CNS) involvement and its relation to pain insensitivity in HSAN4. We present a 15-year-old girl with HSAN4, displaying clinical signs suggestive of CNS impact, including spasticity and a positive Babinski’s sign. Using Technetium-99m ethyl cysteinate dimer single-photon emission computed tomography (Tc−99m ECD SPECT) imaging, we discovered perfusion deficits in key brain regions, notably the cerebellum, thalamus, and postcentral gyrus. These regions process pain signals, providing insights into HSAN4’s pain insensitivity. This study represents the first visualization of CNS perfusion abnormality in an HSAN4 patient. It highlights the intricate relationship between the peripheral and central nervous systems in HSAN4. The complexity of HSAN4 diagnosis, involving potential unidentified genes, underscores the need for continued research to refine diagnostic approaches and develop comprehensive treatments.

## 1. Introduction

Hereditary sensory and autonomic neuropathy type 4 (HSAN4), also known as congenital insensitivity to pain with anhidrosis (CIPA), is an exceptionally rare autosomal recessive congenital disorder. It arises from mutations in the neurotrophic tyrosine kinase receptor type 1 gene (NTRK1) [1,2], responsible for encoding the tropomyosin receptor kinase A (TrkA) [3]. TrkA serves as a high-affinity receptor for nerve growth factor (NGF) [4,5], a pivotal neurotrophic protein crucial for the development, differentiation, and survival of the nervous system [6]. Defects in NGF-TrkA signaling have long been associated with HSAN4, leading to the loss of various NGF-dependent neurons. These include sympathetic postganglionic neurons and NGF-dependent primary afferents, both of which rely on the NGF-TrkA system during their developmental phases [7].

While NTRK1 expression occurs within the brain, the specific role and location of NTRK1 in pain processing for HSAN4 patients remain unclear [8]. Furthermore, the possibility lingers that undiscovered genes may contribute to the complexity of this disorder [9], and the multitude of mutations complicates straightforward DNA diagnosis, rendering it uncommon for routine clinical confirmation of diagnoses [10,11].

HSAN4 manifests with distinctive clinical characteristics, including insensitivity to pain stimuli, absent or severely reduced diaphoresis (anhidrosis), and varying degrees of intellectual disability [1,12,13,14,15]. While several features and pathological aspects of HSAN4 have been partially elucidated [13], limited research has explored the condition of the central nervous system (CNS) in HSAN4 patients. This study aims to investigate whether HSAN4 may affect the CNS, especially considering the presence of upper motor neuron (UMN) signs, often associated with UMN lesions [16]. To address this critical question, we present the case of a 15-year-old Han Chinese teenager diagnosed with HSAN4, who exhibited UMN signs. In our pursuit of understanding the potential CNS impact of HSAN4, we conducted a comprehensive analysis of cerebral blood flow abnormalities using Tc−99m ethyl cysteinate dimer brain perfusion single-photon emission computed tomography (Tc-99m ECD SPECT) brain scan images.

Tc−99m ECD SPECT stands as a pivotal non-invasive diagnostic tool in the realm of neurological assessment [17], primarily chosen for its exceptional ability to evaluate brain blood perfusion. We employed a radiotracer, Tc-99m ECD, which is taken up by the brain’s vascular system and reflects regional cerebral blood flow (rCBF) [18]. SPECT imaging captures gamma rays emitted by the radiopharmaceutical, providing a 3D representation of blood perfusion in the brain. Unlike conventional anatomical imaging methods such as CT or MRI, Tc−99m ECD SPECT provides functional insights into the brain, offering a unique window into cerebral activity. What makes it particularly invaluable is its high sensitivity to changes in blood flow, enabling the detection of even subtle alterations, a feature crucial in diagnosing conditions affecting cerebral perfusion, including stroke, vascular disorders, and neurodegenerative diseases [19].

In stroke assessment, the localization and extent of lesions as a result of defects in blood supply can be assessed via perfusion SPECT studies [20], guiding critical treatment decisions. In cases of dementia, it aids in differentiating between various subtypes [21], contributing to accurate diagnosis and tailored interventions. When it comes to brain tumors, Tc−99m ECD SPECT assists in delineating tumor margins and assessing the viability of surrounding brain tissue [22], providing essential information for surgical planning.

In essence, Tc−99m ECD SPECT’s non-invasive nature, sensitivity to blood flow changes, precise localization capabilities, dynamic imaging capacity, and quantitative analysis options render it an invaluable asset in neurological diagnostics and patient care. Thus, we utilize Tc−99m ECD SPECT to evaluate the brain blood perfusion condition of this 15-year-old Han Chinese girl with HSAN4 with an aim to discover more of this complex disorder.

## 2. Case Description

The subject is a 15-year-old Han Chinese girl, born via cesarean section in 2007. Her developmental journey, however, charted a unique course. Her milestones were delayed, with rolling over achieved at 8 months, sitting at 10 months, and walking at 18 months. Recognizing these delays, her concerned mother sought medical advice, eventually leading to the issuance of a disability card when the patient was just 3 years old. Subsequently, the patient embarked on a rigorous pediatric rehabilitation journey encompassing physical therapy, occupational therapy, and speech therapy. At the age of 6, a seemingly trivial incident cast a spotlight on her condition. She experienced a left radial bone fracture, an event that would typically elicit strong emotional and physical responses. However, in her case, there was a striking absence of any noticeable reaction. This unusual response triggered a series of medical examinations and investigations into the underlying cause. In 2017, driven by clinical suspicion of HSAN4, a polymerase chain reaction (PCR) test was conducted at Changhua Christian Hospital. However, this initial attempt failed to identify any disease-causing mutations. In the same year, she underwent the Wechsler Intelligence Scale for Children-IV (WISC-IV) at Taichung Veterans General Hospital, which unveiled a Full-Scale IQ (FSIQ) of 62, indicating mild mental impairment. The journey for a definitive diagnosis continued. In 2019, next-generation sequencing (NGS) was employed at National Taiwan University Hospital (NTUH), an advanced technique with the potential to uncover elusive genetic mutations. However, the results of this investigation remained inconclusive, underscoring the complex nature of HSAN4. It is worth noting that gene mutations in NTRK1 may not always be directly linked to the clinical manifestation of the disorder. Therefore, a comprehensive clinical evaluation is of paramount importance. Based on her clinical presentation, an expert neurologist at NTUH arrived at a clinical diagnosis of HSAN4.

The year 2020 marked a significant turning point, albeit driven by unforeseen circumstances. The global COVID-19 pandemic necessitated a reduction in the frequency of her rehabilitation sessions. This change had a profound impact on her condition, resulting in a substantial deterioration in her physical and cognitive abilities. To quantify this regression, she underwent a reevaluation with the WISC-IV at NTUH. The results were striking; her FSIQ had plummeted to 52, now indicating moderate mental impairment. Additionally, a battery of neuropsychological tests was administered, including Conners’ Continuous Performance Test-II, which suggested difficulty in maintaining focus, and the Adaptive Behavior Assessment System-II, indicating mild adaptive functioning problems. The impact of the pandemic on her rehabilitative journey underscored the fragility of her condition and the importance of uninterrupted care.

**Figure 1:**
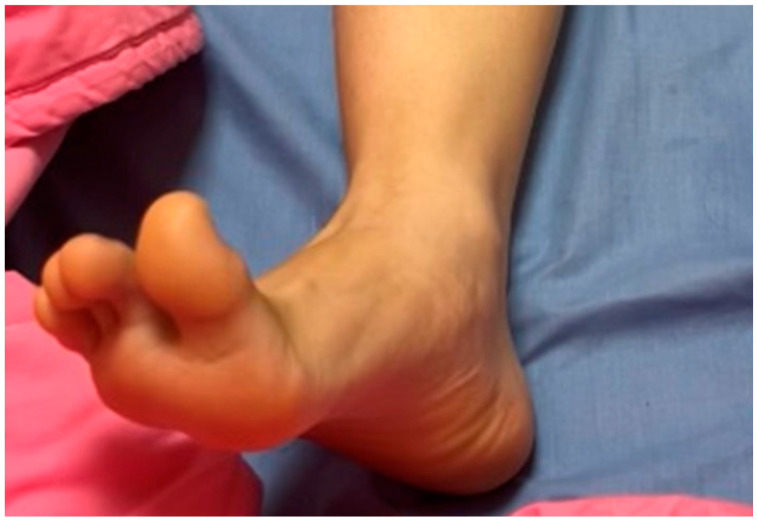
Spasticity occurred in the patient’s feet; this picture was taken when she was sleeping.

In 2022, amidst the ongoing challenges posed by her complex medical condition, she was admitted to the Department of Physical Medicine and Rehabilitation at Kaohsiung Veteran General Hospital for further evaluation. During her hospitalization, two critical observations emerged. First, despite the sultry conditions, there was a conspicuous absence of diaphoresis, further aligning with the classic features of HSAN4. Second, the presence of reduced peripheral nerves was noted in the nerve biopsy. These observations provided additional clinical validation of the HSAN4 diagnosis made by NTUH. Most importantly, the patient showed spasticity in her feet; this phenomenon persisted even during her sleep (Figure 1). During neurological examination, a strong positive Babinski’s sign in both feet was also noted. To discover more into the potential CNS involvement, Tc−99m ECD SPECT imaging was conducted. This imaging technique offers a unique window into cerebral blood flow abnormalities, a key indicator of CNS function. The results of the Tc−99m ECD SPECT scan were both illuminating and thought-provoking.

## 3. Discussion

**Figure 2:**
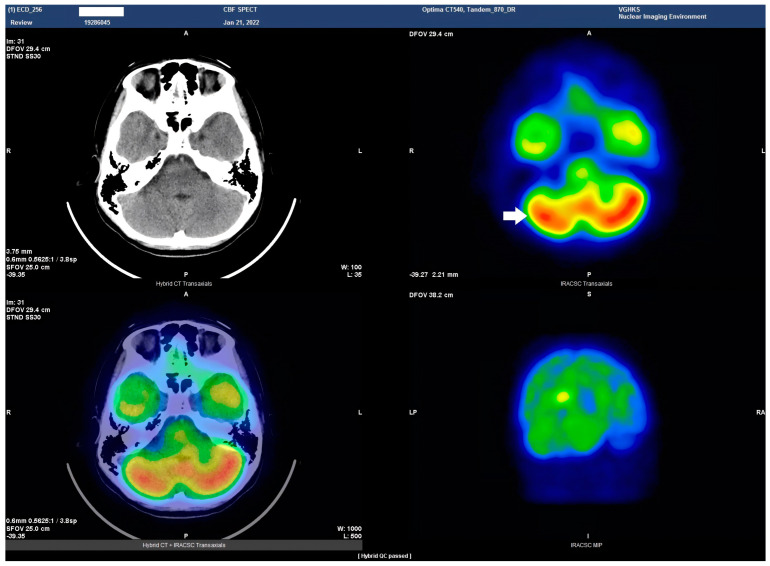
Axial view of the Tc−99m ECD brain perfusion SPECT study. A decreased uptake in the tracer(Tc−ECD) can be observed in the right cerebellar hemisphere (arrow). Left upper panel, CT image; right upper panel, SPECT; left lower panel, the combined SPECT and CT images.

**Figure 3:**
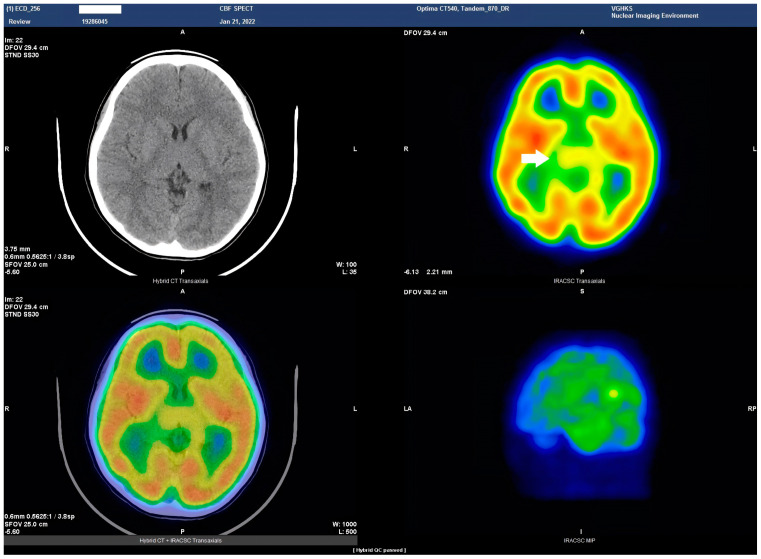
Axial view of the Tc−99m ECD brain perfusion SPECT study. A decreased uptake in the tracer can be observed in the thalamus (arrow). Left upper panel, CT image; right upper panel, SPECT; left lower panel, the combined SPECT and CT images.

**Figure 4:**
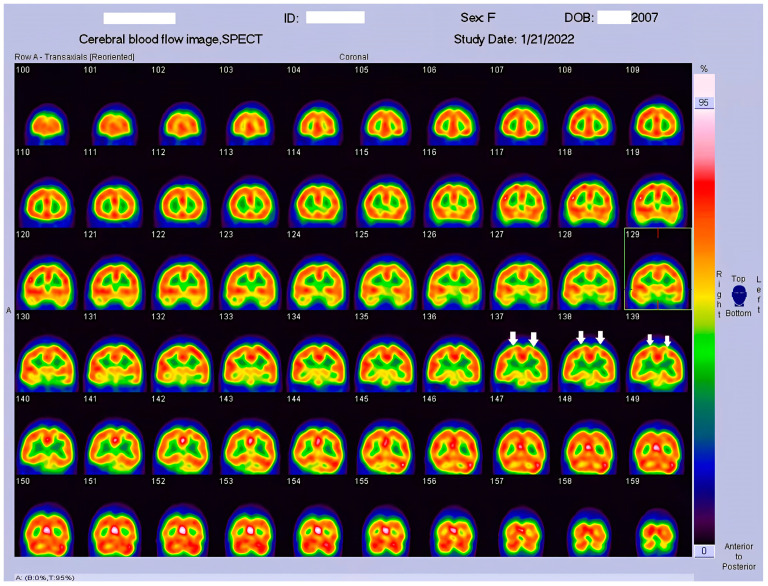
Coronal view of the Tc−99m ECD brain perfusion SPECT images. A decreased uptake in the tracer can be observed in the parietal area, especially in the postcentral gyrus (arrow).

**Figure 5:**
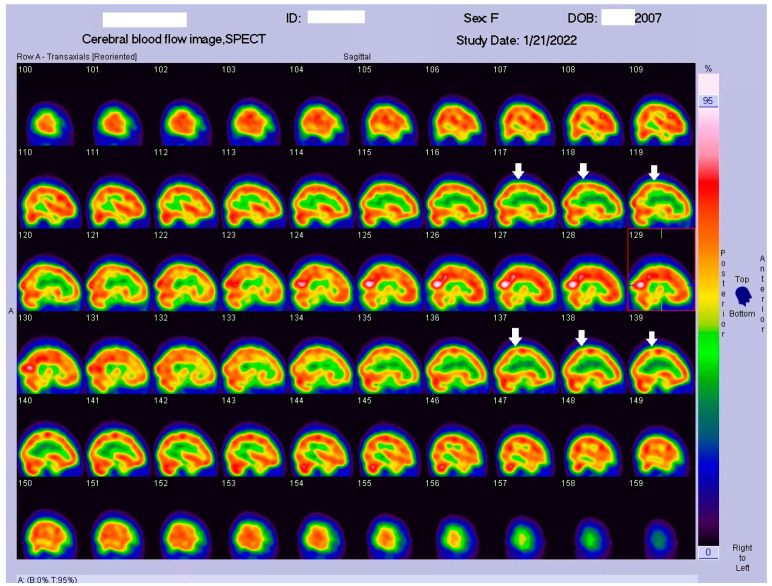
Sagittal view of the Tc−99m ECD brain perfusion SPECT images. A decreased uptake in the tracer can be observed in the parietal area, especially in the postcentral gyrus (arrow).

HSAN4 is a rare genetic disorder with a complex clinical presentation. Our hypothesis that the CNS might be implicated in this condition gained substantial support from the clinical manifestations observed in our patient. The presence of spasticity in her feet and a strong positive Babinski’s sign, which are characteristics commonly found in patients with upper motor neuron (UMN) lesions [16], prompted us to explore the potential involvement of the central nervous system (CNS) in HSAN4. To investigate this novel aspect, we employed Tc−99m ECD SPECT imaging for assessing cerebral blood flow and revealing perfusion deficits within the brain [23]. This study represents the pioneering visualization of brain perfusion abnormality in an HSAN4 patient, and the findings are nothing short of groundbreaking. Our Tc−99m ECD SPECT imaging revealed multiple areas within the CNS with perfusion deficits. Notably, these deficits were observed in the cerebellum (Figure 2), thalamus (Figure 3), and postcentral gyrus of the parietal lobe (Figure 4 and Figure 5). These findings suggest that HSAN4 has the potential to affect not only the peripheral nervous system (PNS) but also the CNS, ushering in a new era in our understanding of this complex disorder. One of the most significant and intriguing aspects of our findings is the localization of these deficits in the postcentral gyrus and thalamus. These regions play pivotal roles in processing pain signals transmitted from the spinothalamic and spinoreticular tracts [24]. The spinothalamic and spinoreticular tracts are the central pathway for nociceptive signals, conveying pain information from the peripheral nervous system to the cerebral cortex. The former pathway originates from the dorsal root ganglia, traverses the dorsal horn of the spinal gray matter, proceeds to the ventral posterolateral nucleus of the thalamus, and finally reaches the posterior limb of the internal capsule. The latter tract also handles nociceptive signals, routing them to the thalamus and postcentral gyrus [25,26]. The perfusion deficits we observed in these crucial pain-processing areas offer a profound and novel perspective on explaining the pain insensitivity characteristic of HSAN4 patients. While previous research has primarily focused on the peripheral mechanisms of pain insensitivity, our study provides compelling evidence that the origin of this phenomenon might extend beyond the peripheral nervous system.

In essence, pain insensitivity in HSAN4 involves intricate CNS mechanisms. The perfusion deficits in the thalamus and postcentral gyrus shed light on how HSAN4 may disrupt these critical pathways, leading to the observed pain insensitivity. This revelation challenges conventional thinking and highlights the importance of considering both peripheral and central components in our understanding of HSAN4.

Another intriguing aspect of our Tc−99m ECD SPECT imaging results was the presence of a perfusion deficit in the right cerebellum, a region not commonly associated with pain processing but known for its diverse functions [27]. Previous studies have suggested that the vermis and deep cerebellar nuclei are associated with pain-related activation [28]. Furthermore, the cerebellum can distinguish between active and passive pain stimuli and is involved in pain anticipation [29,30]. This suggests that the cerebellum may play a cross-modal modulatory role in pain perception [31]. While further research is needed to fully elucidate the role of the cerebellum in pain processing, our findings indicate that this region might contribute to the complex puzzle of pain perception. The cerebellum’s involvement adds an intriguing layer to our understanding of HSAN4 and suggests that the condition’s impact on pain perception extends beyond the classic pain pathways.

However, it is essential to acknowledge the limitations of our study. First, our diagnostic approach primarily relied on genetic testing and clinical symptomatology to identify HSAN4. The involvement of unidentified genes in this disorder poses significant challenges to achieving accurate diagnosis, underscoring the need for ongoing research to enhance diagnostic procedures. Future investigations should explore whether NGF-dependent neurons or other disorder causing factors are located within the perfusion deficit areas, providing further insights into the disorder’s pathophysiology. Second, intellectual disability could potentially result from brain perfusion deficits. However, it is essential to note that intellectual function is typically linked to the frontal lobe [32], a region not directly correlated with the areas highlighted in our study, and the potential variations in CBF in other CNS regions in HSAN4 patients and its correlation with diverse clinical manifestations, including intellectual disability, still need future exploration. Lastly, our study’s sample size is small, focusing on a single patient case. HSAN4 is an exceedingly rare condition, making it challenging to assemble a larger cohort for similar investigations. Collaborative efforts and multi-center studies may be necessary in order to overcome this limitation and provide a more comprehensive understanding of HSAN4’s impact on the CNS.

Building upon the insights gained from this study, future research should explore several critical avenues. First, a broader investigation involving a larger cohort of HSAN4 patients could help validate and expand upon our findings. This would provide a more comprehensive understanding of the extent and variability of CNS involvement in HSAN4. Furthermore, exploring the precise mechanisms underlying the CNS deficits observed in HSAN4 is essential. Investigating the role of NGF-dependent neurons within the perfusion deficit areas could offer valuable insights into the disorder’s pathophysiology. Additionally, functional imaging studies could help elucidate how these deficits affect sensory and motor functions in HSAN4 patients. In conclusion, our study has uncovered a novel dimension of HSAN4 by demonstrating its impact on the CNS, particularly in regions crucial for pain processing. These findings have significant implications for the clinical management of HSAN4 patients and open new avenues for research into the underlying mechanisms of this rare disorder. While challenges and questions remain, our study marks an important step towards a more comprehensive understanding of HSAN4 and its complex pathophysiology.

## 4. Conclusions

By employing Tc−99m ECD SPECT in a 15-year-old HSAN4 patient, we have unmasked significant CNS perfusion deficits in areas crucial for pain signal processing, notably in the cerebellum, thalamus, and postcentral gyrus, offering a profound and novel perspective on explaining the pain insensitivity characteristic of HSAN4 patients. This approach underscores the clinical potential of Tc−99m ECD SPECT in pinpointing specific perfusion deficit regions, thus advancing diagnostic precision in HSAN4 cases. As the first study to visualize CNS perfusion abnormality in HSAN4, it propels us toward more targeted treatment strategies and enhanced patient care, underlining the value of bridging the peripheral and central nervous systems in managing this complex disorder.

## Data Availability

Data are contained within the article.
